# Role of Vitamin D in Cardiometabolic Diseases

**DOI:** 10.1155/2013/243934

**Published:** 2013-02-25

**Authors:** Chaoxun Wang

**Affiliations:** Department of Endocrinology, Shanghai Pudong Hospital, 2800 Gongwei Road, Huinan Town, Pudong, Shanghai 201399, China

## Abstract

Vitamin D deficiency is a highly prevalent condition. Low vitamin D levels have long been associated with bone diseases, such as rickets in children and osteomalacia and osteoporosis in adults. However, it has become apparent in recent years that adequate vitamin D levels are also important for optimal functioning of many organs and tissues throughout the body, including the cardiovascular system. Evolving data indicate that vitamin D deficiency is associated with an increased risk of cardiovascular disease (CVD). Studies have shown that low vitamin D levels are associated with hypertension, diabetes, metabolic syndrome, left ventricular hypertrophy, and chronic vascular inflammation, all of which are risk factors for CVD. This paper reviews the definition and pathophysiology of vitamin D deficiency, clinical evidence linking vitamin D and CVD risk, diabetes and its complications, and metabolic syndrome.

## 1. Introduction

Vitamin D is a collection of fat-soluble steroids, the two important forms of which are vitamins D2 (ergocalciferol) and D3 (cholecalciferol). While vitamin D2 is the dominant form in invertebrates and plants, vitamin D3 is the dominant human form. Vitamin D is a fat-soluble vitamin and, along with parathormone and calcitonin, the active form of vitamin D (1,25-dihydroxycholecalciferol, 1,25 DHCC, or calcitriol) is largely responsible for the regulation of calcium and phosphorus homeostasis in the body.

## 2. Biosynthesis of Vitamin D

**Figure 1:**
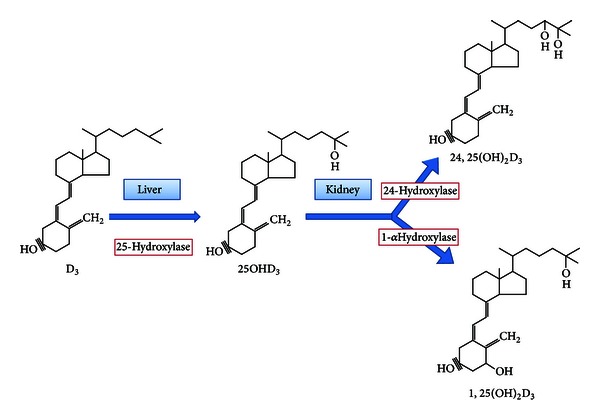
Biosynthesis of vitamin D.

Though vitamin D3 is naturally present in a small range of foods, most of the body stores of vitamin D3 are made endogenously in the skin when 7-dehydrocholesterol, an intermediate in cholesterol biosynthesis, is exposed to ultraviolet B light between wavelengths from 270 to 300 nm. Vitamin D3 circulates in the body bound to vitamin D-binding protein and is rapidly converted to its major circulating form, 25-hydroxyvitamin D (25(OH)D), by the liver. Under the influence of parathormone, 25(OH)D is converted by the 1-alpha-hydroxylase (1**α**-OHase) in the kidney to the active form 1,25 DHCC. A small amount of 25(OH)D is also converted into 24,25 DHCC [1]. Though many other tissues in the body have the 1**α**-OHase and can convert 25(OH)D to 1,25 DHCC, only the renal 1*α*-OHase significantly contributes to circulating 1,25 DHCC levels [2] (Figure 1).

## 3. Vitamin D Receptor

**Figure 2:**
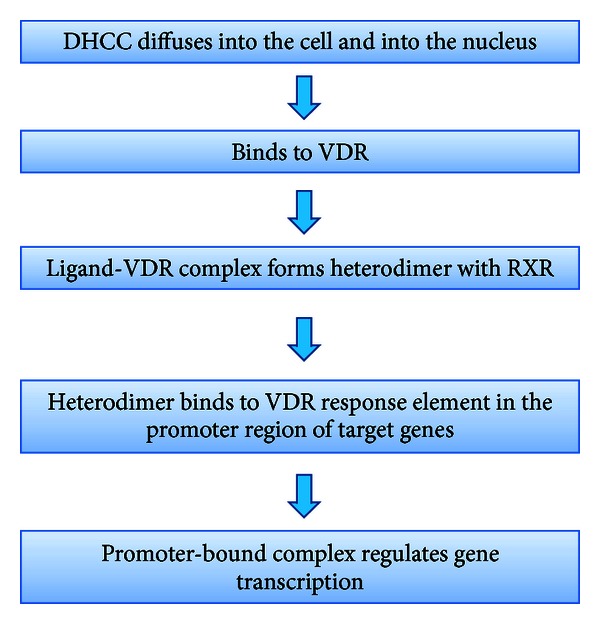
Schematic activity of DHCC in target cells to regulate gene transcription.

Vitamin D is often considered as a hormone rather than a vitamin because of the fact that the active vitamin D metabolite DHCC circulates throughout the body, exerting its wide-ranging effects in cells that contain the vitamin D receptor (VDR). VDR is a type 2 nuclear receptor that is present in the nucleus of cells in most tissues. Circulating DHCC diffuses through the cell membrane and nuclear membrane and binds to the VDR, causing a conformational change in the receptor, leading to its heterodimerization with retinoic acid X-receptor (RXR). This heterodimer then acts as a transcription factor, resulting in expression or transrepression of specific gene products [3]. The entire process has been summarized in Figure 2. It has been estimated that DHCC regulates more than 200 genes, directly or indirectly, thereby influencing a wide variety of physiological processes [3].

## 4. Vitamin D Deficiency

Vitamin D status has been assessed by measuring serum DHCC levels; however, there is no consensus on optimal serum levels or on a uniform assay methodology. Current International Osteoporosis Foundation guidelines define vitamin D insufficiency as 1,25 DHCC levels less than 50 nmol/L (20 ng/mL) and deficiency as levels less than 25 nmol/L (10 ng/mL) [4]. The current consensuses on the serum levels of 1,25 DHCC and the definitions are summarized in Table 1. A 2008 review proposed four categories of Hypovitaminosis D, as summarized in Table 2 [5].

Vitamin D deficiency (VDD) is a well-recognized condition which is prevalent worldwide, particularly at northern latitudes, because of the low levels of ultraviolet B light in winter at these latitudes. Several European studies [6, 7] have shown variation in vitamin D status within countries, which could be explained by factors such as reduced sunlight exposure, low dietary intake of foods rich in vitamin D, limited fortification of food with vitamin D, low physical health status, or differences in biochemical assays used to measure vitamin D levels [8]. A study published in 2001 reported a prevalence of vitamin D deficiency from 2% to 30% of European adults, but found that it increased to 75% or more in institutionalized older persons [9]. Data from the Third National Health and Nutrition Examination Survey (NHANES III) show that approximately one-quarter to one-half of American adolescents and adults are deficient in vitamin D if one uses a threshold of 25 ng/mL [10]. Data from various studies on postmenopausal women revealed that the levels of 1,25 DHCC below 75 nmol/L (30 ng/mL) ranged from 42% in Brazilian women [11] to 92% in South Korean women [12]. Very deficient levels (≤10 ng/mL) are most prevalent in South Asia and the Middle East [13], possibly because of cultural dress that limits sun exposure and extended periods of breastfeeding without vitamin D supplementation.

VDD was thought to be rare in India according to Western view [15]. However, such a belief was based on indirect evidence from studies measuring serum calcium and alkaline phosphatase rather than vitamin D levels. A study published in 2000 established that VDD was present in up to 90 percent of subjects in an Indian city [16]. Later, several studies from different parts of India have established that VDD is very much widespread in Indians of all age groups, including toddlers, school children, pregnant women and their neonates, and adult males and females, residing in both rural and urban areas [17–19]. This high prevalence of VDD in India, despite its sunny climate, is attributed to the skin complexion of Indians, vegetarian food habits, lack of vitamin D food fortification programmes in India, and poor sun exposure [16].

The various causes of vitamin D deficiency can be divided into two broad groups [20]. The first group includes those conditions wherein there is a deficient conversion of inactive vitamin D to DHCC, due to deficient exposure to the UVB component of sunlight. This is commonly seen in people with dark skin, elderly, institutionalized people, immobilized people, and excessive users of sunscreen [21–23]. The second group includes the various medical and physical conditions that can lead to VDD as the followings.

- Drug-induced: long-term use of drugs such as phenobarbital, phenytoin, carbamazepine, rifampicin, and nonnucleoside reverse transcriptase inhibitors (NNRTIs) used in HIV can result in osteomalacia [24–26], probably by inducing metabolism of DHCC.
- Fat malabsorption: since vitamin D is a fat-soluble vitamin, conditions causing fat malabsorption such as Crohn's disease cystic fibrosis and celiac disease can cause VDD.
- Chronic kidney disease: these patients cannot synthesize sufficient 1,25 DHCC from 25(OH)D.
- Obesity: it has been known for a long time that obese people are prone to be vitamin D deficient since they have lower 25-hydroxyvitamin D levels [27, 28]. One explanation was that the subcutaneous fat, which is known to store vitamin D, sequestered more of the cutaneously synthesized vitamin D, resulting in less release of vitamin D from the skin into the circulation in the obese subject than nonobese subject [29].

The various risk factors for developing vitamin D deficiency are summarized in Table 3 [30].

Vitamin D functions to increase serum calcium concentrations through 3 separate activities. First, it is the only hormone known to induce the proteins involved in active intestinal calcium absorption. Furthermore, it stimulates active intestinal absorption of phosphate [31]. Second, it activates resting osteoclasts for bone resorption, thereby mobilizing calcium from bone when it is absent from the diet. It is very important to note, however, that in vivo both vitamin D and parathyroid hormone are required for this mobilization event [32]. Third, both parathyroid hormone and vitamin D interact to stimulate the reabsorption of around 1% of the filtered calcium in the renal tubules. Because 7 g of calcium are filtered every day among humans, this represents a major contribution to the calcium pool [33].

Consequently, the best-known pathological conditions associated with vitamin D deficiency affect bone health. Severe vitamin D deficiency can result in hypocalcemia and hypophosphatemia. This can lead to rickets in children, where the bones are undermineralized as a result of poor absorption of calcium. Similar problems occur in adolescents who are deficient during their growth spurt. Vitamin D deficiency in adults leads to osteomalacia in adults, wherein there is demineralization of bones [34].

## 5. Extraskeletal Role of Vitamin D

After the discovery of the fact that the VDR is expressed in most of the tissues in the body, the role of vitamin D beyond its established role in the maintenance of bone health and serum calcium levels was explored. It was soon found out that vitamin D had several important homeostatic functions not related to calcium homeostasis [2, 31]. The vitamin D receptor is found throughout the body on a number of different cell types, including osteoblasts, pancreatic *β* cells, and nerve cells [35]. Within the cardiovascular system, vitamin D receptors have been found on vascular smooth muscle, endothelium, and cardiomyocytes [36].

Consequently, a growing body of evidence has also accumulated, suggesting that VDD may have extraskeletal manifestations as well. It was also suggested that chronic VDD may have serious adverse consequences such as increased risk of hypertension, multiple sclerosis, cancers of the colon, prostate, breast, and ovary, autoimmune disease, and type 1 diabetes [35]. 

## 6. Cardiometabolic Consequences of Vitamin D Deficiency

**Figure 3:**
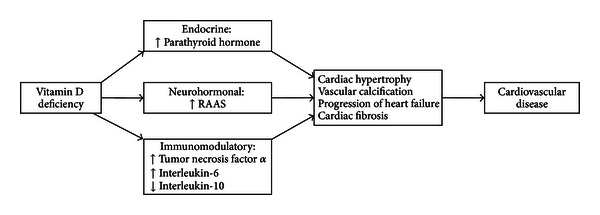
Cardiovascular pathophysiology of vitamin D deficiency.

VDD has been found to contribute to the development of various cardiometabolic conditions such as hypertension, diabetes mellitus, and coronary artery disease. The connection between cardiovascular homeostasis and vitamin D status using a rat model of vitamin D deficiency was first accomplished as early as in 1987 [37]. The evidence of VDD-induced cardiac damage was provided in 1999 in a study done on patients suffering from end-stage renal disease (ESRD). There were studies in which administration of 1,25 DHCC or its analogues to patients with ESRD resulted in decreased left ventricular hypertrophy, along with a decrease in cardiovascular mortality [38, 39], thus proving the involvement of VDD in cardiovascular conditions. A possible mechanism for VDD-induced cardiac damage has been summarized in Figure 3 [40].

## 7. Vitamin D Deficiency and Hypertension

The data from studies on experimental animals linking VDD with increased activation of renin-angiotensin-aldosterone system (RAAS) led to several small studies attempting to correlate vitamin D levels with blood pressures in humans. Though there were significant findings in some of these studies, none of them could establish a strong role for VDD in the pathophysiology of hypertension [41, 42]. 

Subsequently, however, large studies such as the cross-sectional NHANES III and the cohort studies HPFS (Health Professionals' Follow-Up Study) and NHS I (Nurses' Health Study) found an inverse association between serum levels of 25(OH)D levels and blood pressure [43, 44]. Recently, a retrospective cross-sectional study done on 2,722 subjects found out that individuals with serum levels of 25(OH)D less than 40 ng/mL had increased rates of hypertension [45].

This association between VDD and hypertension led to several prospective, randomized, controlled trials studying the effects of vitamin D supplementation on blood pressure. In one of these studies, 148 women aged above 70 years with serum 25(OH)D levels below 20 ng/mL were randomly administered supplementation with either calcium 1200 mg/day only or calcium 1200 mg/day and 1,25 DHCC 800 IU/day. Compared with calcium alone, treatment with 1,25 DHCC led to a significant reduction in systolic blood pressure and heart rate, while having no significant effect on diastolic blood pressure [46]. In another randomized trial which was performed on 34 patients with diabetes mellitus with a serum 25(OH)D level below 20 ng/mL, patients were randomly assigned to receive a one-time dose of ergocalciferol 100,000 IU or placebo. The ergocalciferol group had a significant reduction in systolic blood pressure in addition to the significant improvement in flow-mediated vasodilatation of brachial artery, which was not seen in the placebo group [47]. However, the Women's Health Initiative, in which a combination of low-dose vitamin D3 (400 UI/d) and calcium carbonate supplementation (1000 mg/d) was administered to participants, did not demonstrate an effect on self-reported incident hypertension after 7 years of followup [48].

In 2010, a large systematic review was published in which an analysis of 13 observational studies and 18 randomized trials was made. It noted that while in a meta-analysis of 3 cohorts, lower 25(OH)D concentration was associated with incident hypertension; in another meta-analysis of 10 trials, the supplementation with vitamin D reduced systolic blood pressure nonsignificantly, whereas there was no effect on diastolic blood pressure [49].

Thus, the association between VDD and hypertension is not firmly established. However, in the backdrop of some of the evaluated studies suggesting that VDD may be a risk factor for hypertension, good quality randomized trials are required to elucidate the exact role of VDD in the development of hypertension.

## 8. Vitamin D Deficiency and CVD Risk

Since the activation of the RAAS and the immune system has been linked to vascular disease, exploration of the relationship of VDD with vascular disease was a logical next step. Subsequently, VDD was implicated in several types of vascular disease including peripheral artery disease (PAD), myocardial infarction (MI), coronary heart disease (CHD), and ischemic stroke.

In a NHANES published in 2008, 406 of 4,839 studied patients had PAD (defined as an ankle brachial index of less than 0.9), and these patients had significantly lower mean serum 25(OH)D levels than participants without PAD, although the difference was numerically small. Further analysis revealed a graded association between lower 25(OH)D levels and a higher prevalence of PAD, suggesting that small differences in serum 25(OH)D level can greatly affect PAD risk [50].

A clear association between vitamin D status and the occurrence of acute MI and CHD has not been firmly established. In a study that compared healthy subjects with 128 patients having either acute MI or angina, overall 25(OH)D levels were similar between groups [51]. In another study, 25(OH)D levels between 15 patients with acute MI and 60 age-matched control patients were compared; even here the vitamin D levels did not differ significantly between groups [52]. However, in an analysis of a cohort study, 454 men who reported nonfatal acute MI or fatal CHD had significantly lower levels of 25(OH)D when compared with 900 matched controls without cardiovascular disease; this risk remained significant after adjustment for other risk factors including family history, diabetes, hypertension, race/ethnicity, body mass index, and others [53].

In a cross-sectional study, serum 25(OH)D concentrations were measured at a single outpatient visit of more than 400 diabetic patients but without renal or hepatic disease, and it was found that patients with levels below 20 ng/mL had a higher prevalence of cardiovascular disease [54]. In another study, 1739 participants from the Framingham Offspring cohort were prospectively evaluated to determine if lower vitamin D concentrations could predict the development of cardiovascular disease. A vitamin D concentration below 15 ng/mL was found to be consistently associated with an increased risk of cardiovascular events, and the association retained statistical significance after adjustment for known cardiovascular risk factors including blood pressure, LDL concentrations, renal function, and drug therapy. It was estimated that the 5-year rate of developing a cardiovascular event was two times higher in those with VDD than in those with adequate vitamin D stores [55]. Finally, though Women's Health Initiative found that the supplementation of calcium plus vitamin D did not decrease the risk of CHD or stroke in postmenopausal women, the study had several limitations such as lower dose of vitamin D given to the subjects, subjects in control group also were receiving vitamin D supplements, and nonmeasurement of baseline 25(OH)D levels [56].

Thus, though good quality prospective trials assessing adequate doses of vitamin D supplementation in individuals with relatively low serum levels of vitamin D levels are needed to understand the role of vitamin D in the prevention of cardiovascular disease, it can be safely said that VDD has emerged as an independent risk factor for various cardiovascular diseases including MI and CHD.

## 9. Vitamin D Deficiency and Heart Failure

The fact that VDD causes the activation of the RAAS and the immune system led to the realization that VDD has the potential to cause deleterious effects in patients with heart failure. Subsequently, investigators began to study the association between VDD and heart failure.

Studies performed on patients with decompensated and compensated heart failure and the performance of systolic heart failure patients on the 6-minute walk test showed a non-significant but positive correlation between VDD and heart failure [57, 58].

In a recent study performed on 383 patients with end-stage heart failure awaiting cardiac transplantation, a lower serum 1,25 DHCC values were noted to transform into a higher initial urgent transplantation listing, and lower 1-year survival rates by Kaplan-Meier graph method [59]. In a cohort study, 3299 Caucasian patients who were candidates of coronary angiography were followed up for a median of 7.7 years; those patients with 25(OH)D levels below 10 ng/mL had a significantly increased risk for death due to heart failure and sudden cardiac death when compared with patients with optimal levels of vitamin D, after adjustment for multiple known CVS risk factors. Also, serum 25(OH)D concentrations inversely correlated with the NYHA class [60].

Finally, in a randomized trial evaluating vitamin D supplementation, 123 ambulatory patients with NYHA class II or greater heart failure randomly received calcium 500 mg and either cholecalciferol 2000 IU/day or placebo each day for 9 months. It was found that in the cholecalciferol group, the serum levels of TNF-*α* decreased significantly, and the serum levels of the anti-inflammatory cytokine IL-10 increased significantly. However, vitamin D supplementation was found to be unable to improve survival or alternative markers of heart failure severity such as left ventricle ejection fraction (LVEF), NT-proBNP levels, or C-reactive protein levels [61].

To summarize, the data from the various studies suggest that patients with heart failure have lower serum vitamin D levels. Good-quality randomized trials are the need of the hour to investigate the precise role of vitamin D supplementation for the prevention of development of heart failure in at-risk patients.

## 10. Vitamin D Deficiency and Diabetes

Amongst the various chronic conditions associated lately with VDD, diabetes is perhaps the most important one. 

Various biological factors support the association between VDD and T2DM: (1) studies have found out that the VDR is prominently expressed in both pancreatic beta-cells that secrete insulin and in peripheral target tissues that respond to insulin, such as skeletal muscle and adipose tissue [62, 63]; (2) VDR gene polymorphisms in humans have been associated with variation in insulin secretion and sensitivity [64]; (3) animal studies show that mice with VDR mutations have impaired insulin secretion and poorer glucose tolerance than those with normal VDR [65].

Also, vitamin D has a very important role in ensuring adequate calcium influx through cell membranes, which is required to preserve various insulin-mediated processes in insulin-responsive tissues, such as skeletal muscle and adipose tissue. In support to this finding, an intriguing correlation between vitamin D deficiency and type 1 diabetes has been observed, which may be due to the ability of vitamin D to preserve insulin release modulating the extracellular and intracellular calcium pools.

Further, both T2DM and VDD share similar risk factors, including obesity, physical inactivity (which may correspond with decreased time outdoors and reduced exposure to sunlight), age, and non-White ethnicity. Finally, both glucose levels and serum 25(OH)D vary seasonally. It has been further suggested that the seasonal variation reported for blood glucose may be due to the seasonal variation seen with serum 25(OH)D, which is lower in winter because of decreased sun exposure [3].

Taking cue from these observations, various animal studies performed in as early as the 1980s demonstrated that VDD in rodents and rabbits inhibits pancreatic insulin secretion, indicating that vitamin D is essential for the function of the endocrine pancreas [66]. 

Various cross-sectional studies have shown that patients with type 2 diabetes mellitus (T2DM) have significantly lower circulating concentrations of 25(OH)D, compared to healthy controls [67]. In a study involving septuagenarians who had lower levels of exposure to sunlight, it was found that serum 25(OH)D levels of 50 nmol/L and below were significantly associated with a doubling of risk of developing T2DM. On the other hand, a higher serum 25(OH)D was associated with lower values of HbA1C [68]. Cohort studies have further supported these findings: low baseline 25(OH)D values have consistently predicted the subsequent development of incident T2DM [69, 70].

While observational studies generally support an inverse relationship between serum vitamin D levels and the risk of T2DM, various interventional studies conducted to date have provided inconclusive results. 

For example, in a 2009 study on healthy, middle-aged men with central obesity, vitamin D3 supplementation improved insulin sensitivity [71]. In another study, 81 insulin-resistant, vitamin D deficient women were randomized to receive either 4000 IU of Vitamin D per day or placebo, for 6 months. It was found that the vitamin D group had a significant improvement in insulin resistance, compared with placebo group [72]. A study on east London Asians showed transient improvement of insulin secretion and C-peptide levels in at-risk patients treated with intramuscular vitamin D [73]. In support to these findings, a study in which women with T2DM received 1332 IU of vitamin D3 daily for 1 month showed an improvement on first-phase insulin secretion and a trend toward decreased insulin resistance [74].

On the other hand, a study conducted by Tai et al. found that, in adults without diabetes, correction of vitamin D deficiency was not associated with any effect on blood glucose levels or insulin sensitivity [75]. Thirty-six patients with T2DM on metformin and insulin were randomized to supplementation with either cholecalciferol (40,000 IU per week) or placebo. After 6 months of intervention, fasting glucose, insulin, C-peptide, and HbA1c levels were not significantly different between the two groups [76]. Finally, a study conducted on 61 patients with T2DM concluded that after 16 weeks of therapy, while single high-dose vitamin D therapy showed an improvement in systolic blood pressure and B-type natriuretic peptide levels, there was no difference with regards to insulin resistance of HbA1c levels [77].

However, most of the interventional studies discussed above are done on a small sample size; this might be responsible for the contradictory nature of the results. Thus, well-planned RCTs are required to shed more light on the nature of association between VDD and T2DM.

## 11. Vitamin D Deficiency and Metabolic Syndrome

VDD may be linked with metabolic syndrome by the virtue of some of these observations: (1) vitamin D may blunt the effect of advanced glycation end products on endothelial cells, which contribute to the increased arterial stiffness and endothelial dysfunction observed in individuals deficient in vitamin D; (2) vitamin D may exert protective effects on the vessel walls by inhibition of macrophage to foam cell formation and via its anti-inflammatory effects; (3) vitamin D sufficiency has been associated with downregulation of the renin-angiotensin-aldosterone system [78].

Several cross-sectional studies have reported an inverse association between 25(OH)D and the prevalence of the metabolic syndrome in adults and in children or adolescents [79]. A prospective study involving 524 adults which evaluated the association between 25(OH)D and incident risk of metabolic syndrome reported that baseline 25(OH)D was associated inversely with 10 year risk of metabolic syndrome after adjusting for age, sex, smoking status, season, and body mass index [69]. In another prospective study involving 6,537 Australian adults that was conducted for over 5 years, it was reported that lower 25(OH)D concentrations were associated with not only an increased metabolic syndrome risk, but also a higher waist circumference, serum triglyceride, fasting glucose, and insulin resistance at 5 years [79]. Another recently published cohort study involving 1,801 patients of metabolic syndrome established that optimal serum 25(OH)D levels substantially lowered all-cause and cardiovascular disease mortality in subjects with the metabolic syndrome [78].

All these observations, and the lack of good-quality randomized trials, call for interventional studies that test whether supplementation with vitamin D actually provides a useful adjunct in reducing mortality in these subjects.

## 12. Vitamin D Deficiency and Complications of Diabetes

Various studies have also highlighted the association of VDD with some of the complications of diabetes, most notably diabetic neuropathy and diabetic nephropathy.

Studies have shown that treatment with a vitamin D analogue stimulates the production and prevents depletion of nerve growth factor, which is required for the development and survival of sympathetic and sensory neurons [80]. VDD in patients of T2DM could be a prelude to the development of diabetic neuropathy as the deficiency of vitamin D is known to contribute to the development of neurotrophic deficit [81].

Further, topical [82] and oral [83] supplementations of vitamin D in patients with diabetic peripheral neuropathy have been documented to relieve symptoms and cause a significant reduction in pain scores. Finally, a recently published study again stressed upon the finding that VDD is an independent risk factor for diabetic peripheral neuropathy [84].

While the role of the kidney in the production of the active 1,25 DHCC from the inactive 25(OH)D is established, and as a consequence renal disease has been known to cause VDD, animal studies also suggest that VDD has an active role in the progression of kidney disease [85, 86]. In the kidney, vitamin D may be important for maintaining podocyte health, preventing epithelial-to-mesenchymal transformation, and suppressing renin gene expression and inflammation [87].

Lately, cross-sectional studies have established that there is an independent association between VDD and vitamin D insufficiency with the presence ofnephropathy [88]. A substantial number of studies suggest that replacement with pharmacologic dosages of vitamin D receptor agonists in animal models of kidney disease causes a reduction in albuminuria, abrogation of glomerulosclerosis, glomerulomegaly, and glomerular inflammation [87]. However, with the paucity of randomized controlled studies relating to improvement of diabetic nephropathy with vitamin D supplementation, larger trials are required before any recommendations can be made in this direction.

Finally, that patients with longstanding diabetes are at an increased risk of developing fractures has been an overshadowed association [89]. With the knowledge that VDD is a common factor in the pathophysiology of both osteoporosis and diabetes, good-quality randomized trials are required to see whether supplementation with vitamin D can bring down the rates of fractures in these patients.

## 13. Conclusions

Vitamin D deficiency (VDD) has been classically associated with decreased bone health. However, growing body of evidence has accumulated to suggest beyond doubt that the manifestations of VDD reach well beyond the skeletal system; this is very much apparent by simply observing the wide range of distribution of the vitamin D receptor (VDR).

The role of VDD, along with clinical evidence, in causing and/or aggravating various cardiometabolic disease conditions including hypertension, cardiovascular disease risk, diabetes and its complications such as neuropathy, nephropathy, and diabetic fractures, and metabolic syndrome has been discussed in detail. Despite the presence of numerous studies, both animal and human, the precise role of vitamin D in various cardiometabolic diseases is still a blur. It has been apparent from this paper that in many situations, the results from animal studies, observational studies, and RCTs seem to diverge. Further, in many situations, it is just an observation that VDD and a condition coexist; it is not clear whether the VDD is the cause or the consequence of the condition in question. It might also be possible that vitamin D is not causally related to any health condition but is simply a marker of a general wellbeing and good health. Thus, to clarify the exact role of VDD, good-quality, large, well-designed, and conducted RCTs are still required.

Finally, when it comes to supplementation with vitamin D, to date, the question addressed by intervention studies has been primarily whether vitamin D supplementation may be effective for either the treatment or prevention of various conditions. However, it is important to recognize that all the recommendations regarding vitamin D supplementation have been made keeping in mind the role of vitamin D in bone health. Thus, it is the need of the hour to formulate new guidelines for supplementation with vitamin D, keeping in mind these extraskeletal manifestations of VDD and the potential for the enormous public health improvement that such a guideline may actually bring upon. Till such guidelines are published, it is pertinent to bear in mind the importance of maintaining adequate serum concentrations of vitamin D by means of adequate nutrition and, when indicated or required, supplementation in patients at risk of developing cardiometabolic disorders, in order to achieve better treatment results and improved morbidity and health.

## Figures and Tables

**Table 1:** Defining vitamin D status based on serum 1,25 DHCC levels [4, 14].

Serum 1,25 DHCC levels		Vitamin D status
nmol/L	ng/mL
<25	<10	Vitamin D Deficiency
25–50	10–20	Vitamin D insufficiency
≥50	≥20	Vitamin D sufficiency
>125	>50	Potential adverse effects

**Table 2:** Categories of vitamin D deficiency [5].

Category	Serum levels of 1,25 DHCC	
	nmol/L	ng/mL
Insufficiency	50–100	20–40
Mild deficiency	25–50	10–20
Moderate deficiency	12.5–25.0	5–10
Severe deficiency	<12.5	<5

**Table 3:** Risk factors for developing vitamin D deficiency.

Advanced age
Institutionalized or home-bound
Use of sunscreen with sun protection factor > 15
Heavily pigmented skin
Air pollution
Prolonged, exclusive breastfeeding
Northern latitudes
Smoking
Obesity
Malabsorption syndromes
Renal or liver disease
Antiepileptic or HIV medications
